# Isolated Pectoralis Minor Tendon Rupture with Subclavian Vein Thrombosis

**DOI:** 10.1155/2021/8865592

**Published:** 2021-01-26

**Authors:** Stefan Loske, Mohy E. Taha, Claus Carstens, Kai A. Dietrich, Christian Frank

**Affiliations:** ^1^Division of Orthopaedics and Trauma Surgery, University Hospital of Basel, Spitalstrasse 21, 4056 Basel, Switzerland; ^2^Schulterchirurg, Bahnhofstrasse 24, 5000 Aarau, Switzerland; ^3^Schulterchirurg, Bernstrasse, 84, 4852 Rothrist, Switzerland; ^4^Division of Orthopaedics and Trauma Surgery, Klinikum Mittelbaden, Balger Str. 50, 76532 Baden-Baden Bühl, Germany; ^5^Diagnostic and Interventional Radiology, Klinikum Mittelbaden, Balger Str. 50, 76532 Baden-Baden Bühl, Germany

## Abstract

Isolated insertional ruptures of the pectoralis minor tendon at the coracoid process are a rare condition. Hitherto, very few cases have been reported in the literature. A precise diagnosis is often difficult to obtain and commonly requires advanced imaging to confirm the suspicion and rule out concomitant injuries. All cases reported in the literature to date have been treated conservatively, with excellent results and no further complications. Here, however, we present the case of a patient who had developed a subclavian vein thrombosis. Furthermore, we provide an overview over and draw comparisons to the cases described in the literature. Despite the effectiveness of the conservative treatment, physicians should be aware that adverse events may occur.

## 1. Introduction

Isolated tears of the pectoralis minor tendon are very rare. To our knowledge, only 8 cases have been reported to date [1–7], resulting from two different injury mechanisms: acute traumatic injuries with sudden impact, commonly in contact sport athletes, and atraumatic fatigue ruptures under eccentric loading. The diagnosis is often delayed, and the treatment of choice is traditionally conservative, with excellent outcomes in the literature and no long-term sequelae reported so far.

Here, we present the first case of an isolated pectoralis minor tendon rupture found in a 30-year-old healthy Caucasian male that was suspicious for a deep vein thrombosis of the dominant right arm.

## 2. Case Presentation

As a trained car technician, the work routine of the presented patient consisted of supervising the automatic positioning of the front and rear windows in a large car production line and, in case of imprecise placement, replacing them manually with the help of a coworker. The work sequence included maneuvering a cutting wire between the car window and the car body to loosen the window and subsequently applying suction handles to remove the window. The movement of lifting and handing on the window demands joint abduction in one shoulder, joint adduction in the contralateral shoulder, and anteversion to the horizontal position in both shoulders.

In order to prepare cars for a press conference, he was temporarily transferred to another production plant, and his workload was doubled during this time. Additionally, the intensity was substantially increased because of already hardened connective glue between car windows and car bodies. After the patient had worked under these conditions for two days, he had noticed mild right shoulder pain the following day; however, he had resumed the doubled workload after one day off. During the following working shift, the shoulder pain progressed and he started to notice dysesthesia with restrictions in movements and mild swelling of the affected right arm. Consequently, the patient consulted his general practitioner after finishing the workday, who diagnosed an overuse syndrome of the right shoulder and transferred the patient to our hospital to exclude an arm vein thrombosis.

## 3. Clinical Findings and Imaging

**Figure 1:**
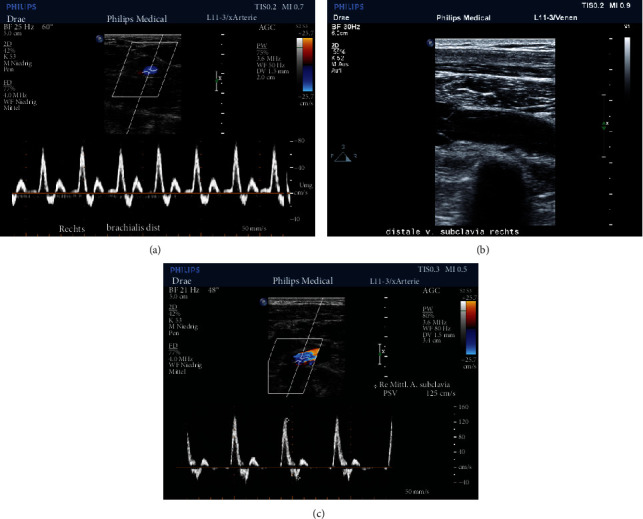
(a–c) Sonography Philips LX 50 transducer L12-3, 12-3 MHz, 160 elements. (a) Brachial artery and vein showing a normal flow signal. (b) Subclavian vein without signs of thrombosis, a venous valve on the left side visible, and no signs of external compression. (c) Subclavian artery and vein. Normal arterial flow, patent vein with no signs of thrombus or external compression.

On clinical examination, the treating emergency physician could objectify neither upper extremity swelling, hematoma, and functional deficits nor prominent lymph nodes in the axillary region. The patient showed no signs of respiratory distress or abnormal thoracic excursions, and auscultation was symmetric. Oxygen saturation was measured 98% under ambient air. Nevertheless, to rule out the suspected diagnosis of the general practitioner, a well-trained specialist performed a Duplex sonography, which revealed normal blood flows and no evidence of thrombosis in both the arteries and veins of the upper right extremity and the subclavian vein (Figures 1(a)–1(c)).

No additional laboratory tests were thus carried out. Symptomatic treatment including nonsteroidal analgesia was prescribed, and the patient returned to work the following morning.

**Figure 2:**
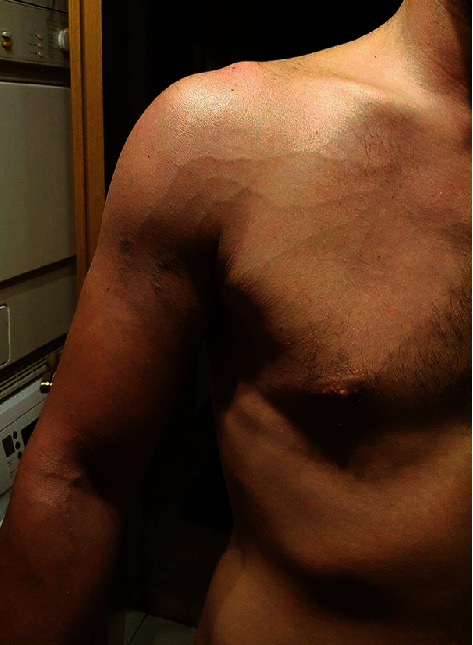
Photo taken by the patient following a minor physical effort five days after the onset of shoulder symptoms showing Urschel's sign and a focal hematoma.

Resuming work aggravated his symptoms, particularly the swelling, over the course of the next two days. Prominent vein signs around his right shoulder became evident, especially after physical effort (Figure 2).

**Figure 3:**
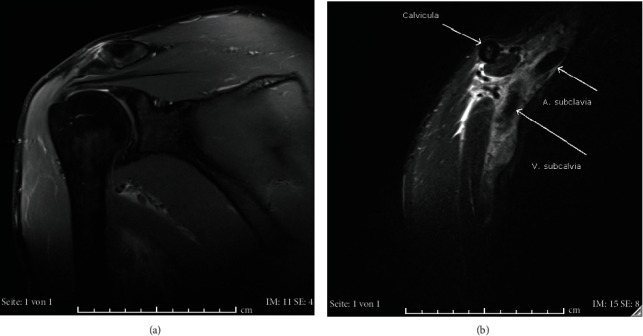
(a, b) MRI of the right shoulder. Siemens Symphony TIM. 1.5 Tesla. Syngo MR B 19. (a) Sequence: PD-TSE coronal, 3 mm TR 2950, TE 31, Acquisitions Matrix 0/384/296/0: shoulder MRI in the coronal plane without pathological findings. (b) Sequence: T2-STIR coronal, 3 mm, TR 6770, TE 35, IR 150, Acquisitions Matrix 0/384/296/0: patent subclavian vein without signs of thrombosis. Of note, MRI conducted in an external institution. Plane has been labelled as coronal, although it appears rather sagittal.

An MRI of the right shoulder was subsequently conducted, revealing a rupture of the pectoralis minor tendon with diffuse hematoma approximating the vessels, but without compression of the vascular lumen. Subclavian vein thrombosis was not diagnosed (Figures 3(a) and 3(b)).

**Figure 4:**
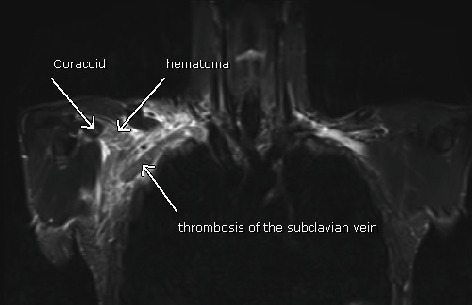
Bilateral chest wall MRI. Siemens Symphony TIM. 1.5 Tesla. Syngo MR B 19. Sequence: T2-STIR TSE coronal, 5 mm. TR 7732. TE 50. IR 140. Acquisitions Matrix: 0/320/244/0: hematoma with suspicion of axillary and subclavian vein thrombosis.

Based on the tendon rupture visualized on MR imaging, the Department of Orthopedics was involved. Symptoms of an arm vein thrombosis seemed to be evident despite the unremarkable imaging studies conducted until then. To extend diagnostics and to use the contralateral shoulder as a reference, an MRI of the entire chest wall was advised 10 days after symptom onset. The MRI was highly suspicious for thrombosis of the subclavian vein (Figure 4).

**Figure 5:**
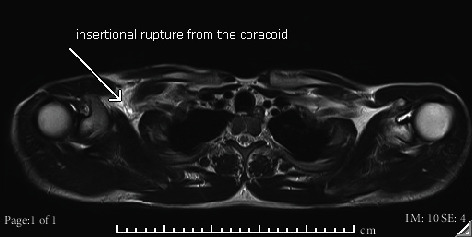
Bilateral chest wall MRI. Siemens Symphony TIM. 1.5 Tesla. Syngo MR B 19. Sequence: T2-TSE transversal, 6 mm. TR 9191. TE 103. Acquisitions Matrix: 512/0/0/179: grade 3 insertional rupture of the pectoralis minor tendon from the coracoid process.

In comparison to the nonaffected left side, a shortening of the pectoralis minor tendon, consistent with a complete insertional rupture from the coracoid process, accompanied by diffuse hematoma with anatomical relationship to both vessels and the coracoid process, was described on the right side (Figure 5).

An additional duplex sonography four days after the second MRI and two weeks after symptom onset confirmed an extensive subclavian vein thrombosis of the right side.

## 4. Treatment

Immediately after the suspected diagnosis of a deep arm vein thrombosis, the general practitioner commenced treatment with Rivaroxaban in a therapeutic dosage (oral administration). The case was thoroughly discussed at an interdisciplinary conference with orthopedic, vascular, and thoracic surgeons as well as angiologists. By consensus, the decision to continue conservative treatment with therapeutic doses of Rivaroxaban was made. Vascular surgeons and angiologists opted against surgery due to the two-week delay of diagnosis [8]. Fibrinolytic therapy was not administered because of the persistent hematoma and time delay [9]. Regular follow-ups with the angiologist were arranged.

Regarding the tendon rupture, a nonsurgical treatment was preferred. Physiotherapy was initiated once follow-up ultrasounds had confirmed a stable blood clot, indicating that the thrombosis was stabilized on the endothelial wall, six weeks after symptom onset. The protocol included glenohumeral centering exercises, isometric rotator cuff strengthening, and shoulder rhythmic stabilization.

## 5. Outcome

One year after the diagnosis, the patient continued to notice intermittent swelling of the right arm and still wore the compression sleeve. He described occasional pain in the right axilla, pronounced in overhead activities and abduction, without signs of an arterial Thoracic Outlet syndrome (no thermic dysregulation, pale hand, or positive Adson-Test), but with a feeling of swelling and tenderness. The patient could not return to his preinjury work level; a replacement occupation in the office with low physical demand was arranged.

The active and passive ranges of motions in the shoulder, elbow, and wrist joints on the affected right side were not limited, as compared to the nonaffected left side (Table 1).

Rough bilateral force measurements (bilateral hand shake) were symmetric. However, as measured at five sites, we found a circumferential side difference of the right and the nonaffected left upper extremity between +0.5 centimeters and +3.5 centimeters (Table 2).

Sensitivity of the lateral upper arm and the volar lower arm to light touch was reduced, without signs of peripheral nerve injuries or plexus irritation. Prominent superficial deltopectoral veins, also known as Urschel's sign, were still visible. Prolonged oral therapy with Rivaroxaban was stopped at twelve months by the angiologist after sonographic follow-up revealed no complete recanalization of the subclavian vein. No prothrombotic risk factors or diseases could be found, especially no genetic or autoimmune disorders.

## 6. Discussion

We present the first case of a patient with an isolated atraumatic pectoralis minor tendon rupture in combination with a subclavian vein thrombosis. In agreement with the previously reported cases [1–7], the tendon lesion was treated conservatively with an excellent functional result. However, the patient continues suffering from a postthrombotic syndrome caused by the subclavian vein thrombosis.

The pectoralis minor tendon is approximately eighteen millimeters wide and four centimeters long at its coracoid insertion [10]. The principal function of the pectoralis minor muscle consists of depressing the coracoid process and drawing the scapula anteriorly and inferiorly towards the thorax, thus moving the inferior scapular angle posteriorly [1]. With the shoulder in a fixed position, the pectoralis minor acts as a respiratory muscle due to its origin on the outer surface of the third through the fifth rib.

Functional limitations are rather marginal in patients with compromised pectoralis minor function, which can be exemplified in patients surgically treated for neurogenic Thoracic-Outlet syndrome. Pectoralis minor tenotomy at the coracoid process is an established procedure with excellent functional postoperative results [11]

Two different causative trauma mechanisms for isolated pectoralis minor tendon ruptures have been reported in the literature to date. The first six published cases describe injuries in athletes after direct trauma. Common among these cases is a direct impact on the shoulder [1, 3, 4] or the arm in different positions, with either a contracted pectoralis minor tendon (anteversion of the shoulder with extended elbow, e.g., blocking drills [6]) or a pectoralis minor tendon under tension (arm in slight abduction, external rotation, and extension [2]). The most recent case published by Vance et al. [7] describes a second injury mechanism: the isolated pectoralis minor tendon rupture was triggered by a static eccentric load in a fatigued muscle during lateral plank exercise with body weight.

Furthermore, overuse insertional tendinopathy of the pectoralis minor muscle has been described as the “bench-presser's shoulder” by Bhatia et al. [12], providing additional evidence that overuse and fatigue likely play a role in the development of pectoralis minor enthesitis; however, partial or full-thickness ruptures have not been sonographically diagnosed in this case series of seven patients.

An undiagnosed chronic development of the tendon tear seems unlikely when considering the large focal hematoma that was evident in our patient on presentation.

We hypothesize that a combination of fatigue and repetitive dynamic overload, which causes entheseal microtraumas, led to an isolated subacute tear. The work sequence consisted of repetitive adduction and abduction movements under an increased load in an anteverted shoulder, requiring constant repositioning of the scapula. The latter matches the key function of the pectoralis-minor muscle-tendon unit [1] and, in case of exertion, presumably represents the pathomechanism leading to tendon injury, similar to the case reported by Vance et al. [7] and the “bench-presser's shoulder” described by Bhatia et al. [12]. In addition, tensile forces of 340–500 Newton were measured using the cutting wire according to a report conducted by an authorized technical expert, which were rated as too high in an ergonomics workplace assessment and surpass the ultimate tensile strength of the pectoralis minor tendon of 177.15 Newton measured by Moinfar and Murthi [10].

What all known cases have in common is a delayed diagnosis, probably due to a combination of low awareness of this very rare injury among physicians, mild clinical symptoms leading to delayed medical consultation, and the protected and deep anatomical position of the muscle beneath the pectoralis major muscle, covering deformities such as tendon retractions.

The tendon injury in our patient was combined with a subclavian vein thrombosis, also known as Paget Schroetter syndrome. Typical key factors like the patient's young age, male gender, and lack of comorbidities match our reported case [13].

Arm vein thrombosis in young patients is most frequently caused by the Thoracic Outlet syndrome. The term describes five different types of compression syndromes at the upper thorax aperture, such as the Thoracic Inlet syndrome (synonymic Hyperabduction syndrome, Pectoralis minor syndrome) [13, 14]. The Thoracic Inlet syndrome is caused by repetitive mechanical compression or angulation of the subclavian vein in over-head movements, typically beneath the first rib or the insertion of the pectoralis minor muscle at the coracoid process [15]. Such hyperabduction movements do occur in the patient's work sequence. The leading etiological hypothesis of these effort-induced thromboses consists of repetitive microtraumas to the vascular endothelium due to mechanical compression [13, 16]. Other subtypes of the Thoracic Outlet syndrome seem improbable in our patient, as no exceptionally prominent structures that could potentially cause mechanical problems were found in the MR imaging.

Anatomically, the subclavian vein is connected to the first rib and the surrounding tissue by the clavipectoral fascia, which also covers the pectoral and subclavian muscles. One key function of this structure is to maintain patency of the lumen of the subclavian vein by preventing breath-dependent collapse due to pressure changes, thus establishing a continuous blood flow in the low-pressure venous system [17].

We hypothesize that the patient initially suffered from reduced subclavian blood flow caused by the hyperabduction mechanism described above and potentially by the hematoma of the pectoralis minor tendon, without immediate formation of a thrombosis. This might reasonably explain the comparatively mild and subjective symptoms of swelling at the first visit and the unremarkable initial Duplex sonography and shoulder-focused MRI. We suppose that the subclavian vein thrombosis formed at a later stage after the patient had returned to work due to ongoing mechanical stress and supposedly expansion of the hematoma. Hereby, progression of swelling leading to Urschel's sign and evidence of thrombosis in further imaging studies (chest wall MRI, second Duplex sonography) became apparent.

The precise role of the perifocal hematoma at the coracoid process remains controversial to us. On one hand, the hematoma might constitute external mechanical compression to the vein, especially if the clavipectoral fascia remains intact, thus limiting the capacity of distribution of the hematoma and consequently increasing the compartmental pressure. However, at the time of the chest wall MRI, no evidence for external vascular compression could be found. On the other hand, a ruptured clavipectoral fascia might not only reduce compartmental pressure but also alter the fascia's function of suspending the subclavian vein. Accordingly, narrowing of the intravascular lumen might occur, leading to a reduction of blood flow during abduction, which represents a risk factor for the development of thrombosis in Virchow's triad [18].

Contribution or predominance of each factor mentioned above cannot be evaluated conclusively. As no interventional or surgical recanalization of the subclavian vein was conducted, it was impossible to detect endothelium thickening as key factor in the initiation of subclavian thrombosis.

In contrast to the tendon lesion, the thrombosis sources ongoing symptoms of swelling typical for a post-thrombotic syndrome [19], despite conservative treatment by compression and Rivaroxaban during twelve months. Superior outcomes are reported for early endovascular fibrinolytic treatment [9, 13, 20]. However, the window of opportunity for a fibrinolytic treatment was already closed once the thrombosis was diagnosed; furthermore, the patient had a history of injury-related bleeding.

Of note, the risk for pulmonary embolism in Paget Schroetter syndrome is substantial, ranging from 7 to 20% [21]. Fortunately, our patient never developed any pulmonary symptoms. This case stresses the crucial role of timely diagnosis, awareness of complications, and early treatment of upper extremity thrombosis.

We believe that similarities concerning the injury mechanism of pectoralis minor tendon insertional ruptures and Paget Schroetter syndrome and the close anatomical proximity of these two structures represent the principal reason for their simultaneous incidence. Both entities are prone to repetitive abduction movements of the shoulder joint.

## 7. Conclusion

We present a novel case of isolated pectoralis minor tendon in an otherwise healthy nonathlete patient with extensive focal hematoma after repetitive shoulder abduction, likely contributing to subclavian vein thrombosis. The aim of this case is to demonstrate etiological similarities between insertional pectoralis minor tendon ruptures and Paget Schroetter syndrome and to raise awareness for the fact that highly relevant complications in isolated pectoralis minor tendons may occur. Extensive attentiveness of the treating physician is essential to detect subclavian vein thrombosis promptly, in order to prevent both catastrophic short-term complications such as pulmonary embolism and long-term complications such as postthrombotic syndrome.

## Figures and Tables

**Table 1:** 

Active range-of-motion of bilateral glenohumeral joints (in degrees)		
	Left	Right
Abduction/adduction in neutral rotation in the scapular plane	150-0-40	150-0-40
Internal rotation/external rotation in 0° glenohumeral abduction	90-0-70	90-0-70
Internal rotation/external rotation in 90° glenohumeral abduction in the coronal plane	70-0-90	90-0-90
Anteversion/retroversion in neutral rotation in the scapular plane with the scapula fixed	110-0-60	110-0-60

**Table 2:** 

Circumferential measurements of bilateral upper extremities (in centimeters)		
	Left	Right
10 centimeters above radiocapitellar joint line	31.5	32
At the level of radiocapitellar joint line	28	28.5
10 centimeters below radiocapitellar joint line	26	29.5
At the level of radiocarpal joint	17.5	18
At the level of diaphyseal metacarpal bones	22	23

## Data Availability

The data used to support the findings of this study are included within the article.
